# The Future of Aging: Transforming healthcare for Nigeria’s Aging Population

**DOI:** 10.1093/geroni/igaf122.449

**Published:** 2025-12-31

**Authors:** Chinenye Aniemeke, Joy Demesi, Ebi Ofrey, Ajibola Meraiyebu, Gregory Orewa

**Affiliations:** The University of Texas at San Antonio, San Antonio, Texas, United States; The University of Texas at San Antonio, San Antonio, Texas, United States; GeroCare Solutions Ltd, Lagos, Lagos, Nigeria; Gerocare, Abuja, Federal Capital Territory, Nigeria; The University of Texas at San Antonio, San Antonio, Texas, United States

## Abstract

Nigeria’s healthcare crisis disproportionately impacts its growing older adult population, now exceeding 10 million. The healthcare crisis has been exacerbated by the migration of skilled healthcare professionals leaving the country. Limited geriatric care, high healthcare costs, and lack of structured home services leave many individuals with undiagnosed chronic conditions and preventable complications. GeroCare, an innovative solution, addresses this gap by offering home medical visits, teleconsultations, and caregiver support through a network of physicians. This model utilizes technology to enhance accessibility and improve care coordination. This study explores how GeroCare impacts healthcare access, quality of life, and family caregiving experiences for older adults in Nigeria, as reflected in patient testimonials. This study employs thematic analysis to identify recurring patterns from qualitative data from patient testimonials collected between 2017 and 2024 in written, audio, and video formats. Key themes included increased healthcare access, enhanced quality of life, and improved peace of mind for families. Findings indicate that GeroCare improves access to care for the aging population and relieves caregiving burdens. The study emphasizes technology-driven home healthcare models to fill systemic gaps in older adult care. It highlights the need for policy reforms to integrate telehealth into national healthcare frameworks and expand insurance coverage for home-based medical services across low-resource settings. Additionally, scaling models like GeroCare can alleviate workforce shortages by leveraging telehealth, task shifting, and remote monitoring to optimize healthcare delivery. Policymakers, healthcare providers, and stakeholders should consider an innovative model like GeroCare to improve healthcare for older adults in low-resource settings.

